# What clinic closure reveals about care for drug-resistant TB: a qualitative study

**DOI:** 10.1186/s12879-023-08405-7

**Published:** 2023-07-17

**Authors:** Thiloshini Govender, Jennifer J. Furin, Alex Edwards, Selvan Pillay, Richard A. Murphy

**Affiliations:** 1 King Dinizulu Hospital Center, Durban, South Africa; 2 Harvard Medical School, Cambridge, MA USA; 3 Emory University School of Medicine and Rollins School of Public Health, Atlanta, GA USA; 4 University of Lincoln, Brayford Pool, Lincoln, UK; 5 Adrenergy Research Innovations, Durban, South Africa; 6 Geisel School of Medicine at Dartmouth, Hanover, NH USA; 7 White River Junction Veterans Affairs Medical Center, Medicine Service 163 Veterans Drive, 05009 White River Junction, VT USA

**Keywords:** Qualitative research, Patient-centered care, Drug-resistant tuberculosis, Multidrug-resistant tuberculosis, Rifampin-resistant tuberculosis

## Abstract

**Background:**

There have been calls for “person-centered” approaches to drug-resistant tuberculosis (DR-TB) care. In 2020, Charles James Hospital in South Africa, which incorporated person-centered care, was closed. Patients were referred mid-course to a centralized, tertiary hospital, providing an opportunity to examine person-centered DR-TB and HIV care from the perspective of patients who lost access to it.

**Methods:**

The impact of transfer was explored through qualitative interviews performed using standard methods. Analysis involved grounded theory; interviews were assessed for theme and content.

**Results:**

After switching to the centralized site, patients reported being unsatisfied with losing access to a single clinic and pharmacy where DR-TB, HIV and chronic disease care were integrated. Patients also reported a loss of care continuity; at the decentralized site there was a single, familiar clinician whereas the centralized site had multiple, changing clinicians and less satisfactory communication. Additionally, patients reported more disease-related stigma and less respectful treatment, noting the loss of a “special place” for DR-TB treatment.

**Conclusion:**

By focusing on a DR-TB clinic closure, we uncovered aspects of person-centered care that were critical to people living with DR-TB and HIV. These perspectives can inform how care for DR-TB is operationalized to optimize treatment retention and effectiveness.

## Background

Despite the existence of curative therapy for tuberculosis (TB) since the 1950s, TB treatment has historically been driven by a paternalistic “public health approach” and it remains one of the leading infectious killers of adults worldwide [1]. This is especially true for drug-resistant forms of TB (DR-TB) that are characterized by strict models of service delivery, emphasis on a “one-size-fits-all” approach, and poor outcomes, with success rates of just over 60% reported globally [2]. Recently, there have been calls for more “person-centered” approaches to TB and DR-TB care which are grounded in a different, human rights model of service delivery [3]. Agreement exists that person-centered services are characterized by TB care that is holistic, individualized, empowering, and respectful [4]. Moreover, person-centered care is associated with high rates of DR-TB treatment completion, improved retention in care and appropriate management of concomitant HIV infection [5].

One aspect of DR-TB treatment that has made it challenging to incorporate elements of person-centered care is that services have been highly centralized, usually at specialized medical centers in selected areas of a county [6]. While this centralized model of treatment delivery understandably emerged from a lack of clinical expertise in management of people living with DR-TB and from the need to ease provision of services from a health systems point of view, its persistence is associated with barriers to care and catastrophic costs [7]. In contrast, multiple studies have found that decentralized DR-TB care is both a cost-effective – for the health care system and for people living with DR-TB – and the preferred means for delivering DR-TB therapy [8, 9]. Decentralized DR-TB services are recommended by the World Health Organization (WHO) for the majority of people living with DR-TB, and most countries include a plan for decentralized DR-TB care in national guidelines [10].

## Methods

In 2011, South Africa introduced an ambitious plan to decentralize treatment for DR-TB to minimize delays prior to treatment initiation and allow for DR-TB treatment closer to where patients live [11]. One trigger for this shift was a rapid rise in the number of newly identified patients with drug resistance – after the wide introduction in South Africa of the Xpert MTB/RIF assay – which rapidly increased referrals of people living with DR-TB to centralized tuberculosis hospitals [12]. In 2016, Charles James TB Hospital became one such decentralized site where patients who resided in the catchment area of the hospital south of Durban were hospitalized to initiate DR-TB therapy and then followed monthly at an on-site clinic where antiretroviral therapy (ART) was also provided. Charles James became the location of a cohort study, “Optimizing Adherence Support Inspiring Success” (OASIS), that sought to study adherence to DR-TB therapy and ART across the 9-month short-course regimen; it was the parent study and qualitative interviews were a planned component. When the hospital was suddenly closed in 2020, patients were referred mid-course to a more centralized, tertiary hospital 13 km away to continue treatment. This unanticipated closure provided an opportunity to examine person-centered care from the perspective of patients who lost access to it and experienced “recentralization,” and we report the results of a qualitative exploration of this experience.

### Setting

In 2018, South Africa revised its guidelines for the treatment of patients with DR-TB, which included replacing the injectable agent kanamycin with bedaquiline [13]. This short-course, fully-oral regimen consisted of bedaquiline, linezolid (for the first 2 months only), levofloxacin, clofazimine, high-dose isoniazid, ethambutol, and pyrazinamide for the first 4–6 months followed by levofloxacin, clofazimine, ethambutol and pyrazinamide for the remaining 5 months. This regimen was rolled out to decentralized treatment sites including Charles James where patients were hospitalized – initially for the first 2–3 months but for a shorter period after national policy revisions – and were discharged to follow-up monthly at the on-site clinic.

At this decentralized site, key elements of person-centered care were integrated into the program. The hospital and clinic were located on a small, uncrowded campus, geographically proximate to patients. There was on-site HIV treatment fully integrated with the DR-TB clinic (both HIV and DR-TB was addressed by a single medical provider during one medical visit) together with a single pharmacy providing ART and DR-TB medicines. After inpatient hospitalization, patients received community-based adherence support for DR-TB treatment rather than strict DOT. Medical care from month to month was provided by a small, consistent team of physicians and nurses. The waiting times at the clinic and pharmacy were short. Patients who missed a clinic appointment were contacted the following day to avoid treatment interruption. Nursing staff engaged patients consistently at monthly visits on adverse events and adherence. Deficits in disease and treatment literacy were addressed. A social worker was actively engaged in psychological assessments and counseling, and assisted patients in applying for financial support.

### Qualitative methods

The impact of the closure of this decentralized DR-TB site was explored through series of open-ended, qualitative interviews done with purposively selected individuals. These interviews consisted of questions and probes aimed at understanding the experience of the participant with hospital closure [14]. The interviewer, a female nurse, underwent training in qualitative interviewing through the South African Medical Research Council and, prior to actual interviews, participated in mock sessions.

The goal of these interviews was to understand the ways in which care was transitioned during the clinic closure and to hear from individuals receiving care for DR-TB what their experiences with treatment were like during this time. Interviews were performed in person in a research office at Prince Mshiyeni Memorial Hospital in the language preferred by the study participant (either isiZulu or English), recorded and transcribed into English for analysis. Transcripts were not returned to the participants and they did not review the coding, analysis, or final manuscript. Interviews lasted between 15 and 60 min. Separate field notes were not recorded. The only people present at the interview were the participant and the interviewer. Repeat interviews were not carried out. Transcripts were not shared with participants and participants did not provide feedback on findings. Data analysis involved grounded theory with interviews assessed for theme and content [15]. An initial coding scheme was developed by one study team member (JF) who is trained in qualitative research and has significant experience with qualitative studies in infectious diseases. This scheme was shared with the other study authors and modified based on their input. Themes and patterns were assessed using the four domains of person-centered services (holistic, individualized, empowering, and respectful care). Study data were collected and managed using REDCap electronic data capture tools [16, 17].

### Mapping analysis

The data from these open-ended interviews was supplemented with a mapping exercise performed to detail potential geographic and travel barriers that may have been introduced into care during recentralization. Spatial statistical analysis was performed using ArcGIS 10.8 (ESRI, Redlands CA) in addition to Google Maps (Alphabet, Mountain View CA) for geocoding. Forty-nine participants, comprising the overall OASIS study cohort, had valid addresses and were included in this analysis, seven were excluded. Each participant’s home location was masked to maintain privacy on an individual basis with their latitude and longitude jittered by a random offset of up to 150 m to a nearby commercial business. Each hospital was assigned an exact location as presented on the KwaZulu-Natal Provincial Department of Health web page [18].

Distance in meters to care from home location of each participant to each hospital was calculated using Manhattan distance along the South African road grid provided by The World Bank [19]. These two distance’s means were then compared in RStudio (Boston, USA) using a paired t-test.

### Human subjects

The study was approved by the Biomedical Research Ethics Committee at the University of KwaZulu-Natal in Durban (BE130/19) and at the University of California, Los Angeles and informed consent was obtained from each participant. All methods were carried out in accordance with relevant guidelines and regulations.

## Results

A total of 57 persons received care for DR-TB at Charles James Hospital during the study period. Our goal was to interview between 5 and 10% of these individuals for the qualitative study. We selected 6, all of whom agreed to participate. These interviews were analyzed as described in the methods section, and after the fourth interview was analyzed, no more than 5% new information was uncovered in interviews 5 and 6. Thus, we considered that saturation had been reached according to the methods of Guest et al [20]. The demographics of the participants are described (Table 1). In the domain of services that are holistic, sub-themes included longitudinal care and care for co-morbidities. In the domain of individualized care, sub-themes included choice and manner of referral to the new health care facility as well as understanding of individual needs and barriers to services. In the domain of empowering care, sub-themes included knowledge of providers and communication around health issues. In the domain of respectful care, sub-themes included stigma/discrimination and perceptions of service crowding and waiting times. Each of these are explored in more detail below, along with selected quotes from study participants. Overall, the study revealed that – while there were some areas in which person-centered care was achieved after transition – the new, larger clinic was not experienced as person-centered in most respects (Table 2).

### Holistic care

The most notable aspect of care at the new site that was not holistic was the fact that people living with HIV and DR-TB could not receive their DR-TB treatment and antiretroviral therapy (ARVs) from the same provider or at the same visit. Participants reported having to wait in multiple queues for different types of medical treatment and medications. This lack of integrated services was noted by several participants, as shown in the quote below:

*You had to wait very long in a queue and if bloods were taken you have to wait for your results first prior to getting your meds and at the pharmacy you have to wait again for longer whilst you still have to wait at ARV clinic for your ARVs meanwhile at [Charles James Hospital] we had to get all our meds at once no long queues. Why can’t they give us all our meds at once both ARVs and TB meds?*

They also reported a lack of integrated services for other co-morbidities in addition to HIV, including COVID-19.

Participants also felt that being able to see providers who knew them over longer periods of time contributed to care that was holistic in nature, in large measure because the providers understood other aspects of the participants lives. They were reassured during the transition to the new hospital when they were able to see the same provider, as described by one participant.

Fortunately, I came across my doctor from [the previous health center] at [the new health center] which made me happy and he continued where he left off.

A lack of continuity of care, however, was described as a serious challenge in the new health center, where DR-TB care was provided by multiple providers. As one participant reported:

*At [the previous health center] we had one doctor and at [the new health center] I was seen by different doctors and that was putting a lot of strain on me cause it’s difficult for a doctor to know if whatever problems you had previously was addressed and know if there are any improvements. Now you had to explain even to this new doctor what you had been given maybe is not helping you.*

### Individualized care

In terms of services that were individualized, participants reported care that was not person-centered. This was manifest by not being given a choice about the closure of their DR-TB facility or a choice about where they could receive new treatment services. As one participant reported:

*I was worried as to how will the new hospital treat me since I’ll be new and the thought of waiting at the hospital for long was really stressing me. But hey I had no choice but to go there*.

Participants also reported that there was no consideration of the individual barriers they would face to receive care, including transportation costs and time. This was described by one participant below:

*I wish there was something arranged or organized for us so that it’s easy for us to get help and also some of us didn’t even know how to get to [the new clinic] and you end up getting lost and lost and spending a lot of money*.

### Empowering care

In terms of empowering care, participants did report they felt the care was empowering in some ways, especially when the providers were seen as knowledgeable and were willing to share that knowledge with the participants. As one person reported:

*I just trusted them because they know what they are doing.*

There were aspects of care, however, at the new facility that were felt not to be empowering. Several of these had to do with a lack of communication with participants, especially around test results. One participant also reported that she felt her care needed to be improved because the staff kept losing her files. This experience was demoralizing for her, as described below:

*The doctor was okay but what use to stress me was that my file was always missing and a new one has to be made. Which makes it difficult for the doctor to know how I was or what I complained of previously. 3 files of mine went missing and I had to get a new one each time.*

### Respectful care

Participants reported feeling stigma and discrimination at the new facility and felt this took away from the quality of care their received. They contrasted this with the care they received at the previous center, where they were treated in a respectful and humanistic manner. This is illustrated in the quote from one participant below:

*Health workers should not make MDR patients to be kept away or on isolation cause it means you are treated in a different way compared to other people. Also my wish is for MDR patients to have a special place for them as it feels so bad when we go to ordinary hospitals and get treated differently and we might feel better if we at least have our own hospital like [the previous health center]. People are so scared of us hence we need to have a place like [the previous health center].*

And another, when speaking about the care received at the previous health center reported:

*We were treated in a special way and we felt very special compared to where I am now*.

Participants also felt that the long waiting times at the new clinic took away from the care experience. As one participant reported:

*Even if you came at 5am you’ll end up in long queues and leave around 1pm even if you happen to be at the pharmacy … there’s even another long queue there… you can’t be at the hospital around 5am till 10 am you waiting and still have to wait another 2 hours at pharmacy? It’s so annoying.*

In addition to waiting times, participants reported that the new facility was crowded and that this also took away from the experience of care they received there. As one reported:

*There is nothing that can be done because [the new health center] has a large number of clients. At [the previous health center] there’s a place for males and place for females but [the new health center] doesn’t have but simply because it is overcrowded.*

### Spatial analysis

After hospital closure and care transfer to the tertiary center, the relationship between place of residence and DR-TB treatment site was explored. After the change, the mean distance to care was reduced by 2 km from approximately 13 km to 11 km; however, this difference was not significantly different *(p < 0.13*, 95% CI (-1448, 662 m)).

## Discussion

There are limited data that reveal areas of service delivery that need to be improved to achieve person-centered DR-TB care. By focusing on an event in which a person-centered DR-TB clinic was closed—largely because the TB program wanted to focus on efficiency—and exploring this event through a series of qualitative interviews and a mapping exercise, we were able to uncover a number of aspects of care that were important to people living with DR-TB, including: (1) holistic care where integrated services for multiple health problems can be delivered longitudinally without the need to attend multiple appointments and wait in multiple queues; (2) individualized care where choices are offered about when and where services are delivered and there is an understanding of barriers faced by people living with DR-TB; (3) empowering care where knowledgeable providers partner with people living with DR-TB throughout ongoing communication and interactions; and (4) respectful care that is attentive, not stigmatizing and inconsiderate. These needs may appear to contrast with those of a public health program—where cost and resource considerations appear to be more significant than the quality of services delivered. However, it is worthwhile considering that it is the sum of individual experience that make a public health program successful (or unsuccessful), and efforts to improve the quality of care are therefore warranted.

As a shorter and more effective regimens for DR-TB are rolled out, we can increase the likelihood that these regimens will succeed for patients in programmatic settings by addressing – with person-centered care – the critical social issues and comorbid conditions that surround DR-TB [21]. One of these is HIV which, in DR-TB programmatic settings (including Charles James), is typically clinically advanced (CD4 < 200 cells/mm^3^); frequently patients living with HIV are diagnosed with DR-TB when ART naïve or experiencing ART treatment failure [22, 23]. The prevalence of HIV is particularly high in southern Africa where 50–70% of patients with DR-TB are also living with HIV. The use of ART is associated with a significant reduction in mortality during an episode of DR-TB [24]. DR-TB treatment sites that integrate HIV care and monitoring into routine services – compared to programs that require additional clinic, lab and pharmacy visits for HIV treatment and monitoring– are well-positioned, in patients living with both conditions, to optimize both HIV and DR-TB outcomes [5, 25]. In interviews, patients living with HIV and DR-TB made it clear that incorporating HIV services in DR-TB treatment programs is a priority and TB programs are urged to diagnose, treat and monitor HIV and other common chronic medical conditions as part of the standard care model.

This qualitative study suggests that decentralized DR-TB care, compared to treatment delivered in larger centralized centers, may be more compatible with the person-centered model. However, in pushing to expand decentralized DR-TB care, advocates must contend with notions that that the centralized model of treatment delivery is “efficient” and “necessary,” in part because supporting multiple decentralized sites appears costlier up front and because clinical expertise in the management of DR-TB has historically not been widely available. However, undermining these assumptions are the excellent clinical outcomes achieved in small, decentralized sites that have included elements of person-centered care [26]. Part of the larger decision-making calculus should be the costs of treatment failure, loss to follow-up, and ongoing transmission of DR-TB when it is posited that decentralized, patient-centered DR-TB care is unaffordable. Beyond cost, a wider consensus has emerged that the person-centered approach is a more ethical approach to care. Consistent with this, in 2001 the influential Institute of Medicine urged movement towards a “partnership among practitioners, patients, and their families (when appropriate) to ensure that decisions respect patients’ wants, needs, and preferences and that patients have the education and support they need to make decisions and participate in their own care,” a recommendation echoed by experts in the field [27, 28].

South Africa has made considerable progress in decentralizing DR-TB care but person-centered treatment principles have not been emphasized as a core component of these efforts. A 2019 South Africa guideline reinforced a national commitment to decentralization DR- TB management, but only briefly referred to person-centered principles [29]. The guidance recommended that treatment be “dictated by the patient’s needs” care and that co-existing HIV be diagnosed and treated but did not emphasize that person-centered principles be a critical part of an effective decentralized treatment framework. Guidance for clinic-level implementation of person-centered care is available and begins with efforts, including structured interviews with patients, to assure that patient needs – not just TB program priorities – become a central concern. One practical set of tools was produced by US Agency for International Developmenrt and the Royal Tropical Institute [30].

In the spatial analysis, we found that – in contrast to our hypothesis – distance from place of residence to treatment site after recentralization did not increase. Despite this, other elements of care clearly altered patients’ perceptions of how well they were being served by the system. We do caution that the spatial analysis is limited given that we do not know the type of transportation participants utilized for travel (for example, taxi, public transport, or private vehicle) which may have made the new location more or less difficult to reach. However, putting distance in a broader context, it may be a mistake to focus on proximity as the most important marker of person-centered care, as other elements appear to be more important to patients than distance alone.

The study had several strengths. The analysis was based on interviews with patients who historically are at particularly high risk for poor DR-TB outcome (80% were living with DR-TB and advanced HIV) making the findings especially relevant for policymakers. A second strength is that patients were part of a known cohort of patients living with DR-TB which – in contrast to some qualitative research – provides an objective measure that we are studying the appropriate patient population. An additional strength is that we also included geospatial analysis which helped us understand that distance was the not the most critical factor in patients’ care experience. The limitations include a relatively small sample size, the inclusion of a patient sample that was largely male and the potential lack of generalizability to other settings with different DR-TB treatment structures.

The experience of patients affected by the closure of Charles James TB Hospital may have particular relevance in the current COVID-19 pandemic era. Although all the factors that precipitated Charles James’ closure are not known, the closure occurred early in the pandemic when there was a reduction in DR-TB case detection and treatment initiation in South Africa – as well as globally – resulting from travel restrictions, changes in health-seeking behavior, disruptions in diagnostic and curative DR-TB services [31–33]. One missing consideration appears to have been how these disruptions would play out in individual lives, perspectives that are documented here. While urgent response to new epidemics are essential, these responses should not compete with ongoing priorities such as diagnosing and treating DR-TB and HIV which in southern Africa are ongoing health emergencies. Nonetheless we are left with the likelihood that the pandemic and resource reallocation will have an effect on TB and DR-TB mortality for years to come [34].

## Conclusion

In conclusion, this study reveals important elements of person-centered care for DR-TB that should be incorporated into service delivery. Without such a focus, advances in newer diagnostic and treatment modalities will not likely have the desired impact on the global TB pandemic. As countries and programs are pushed to maximize short-term efficiency, it is imperative that vulnerable aspects of service delivery, which focus on the individual, be protected and prioritized as essential elements of care.

**Table 1:** Demographics and baseline characteristics of patients participating qualitative study

Characteristics	N = 6	% / Median	95% CI / IQR
Gender			
Male	5	83.3	44.2—98.1
Age, median (IQR)		29.6	26.3—35.4
Education			
Did not complete secondary school	2	33.3	7.7—71.4
Completed secondary school	4	66.7	28.6—92.3
Employment			
Unemployed	6	100.0	67.0—100.0
Monthly income, median rands / month (IQR)	6	0	
Food security, food available every day (last 7 days)			
Yes	6	100.00	67.0—100.0
Living situation			
With parents	3	50.0	16.7—83.3
Other	3	50.0	16.7—83.3
Marital status			
Single	3	50.0	16.7—83.3
Married	3	50.0	16.7—83.3
Tobacco			
Active	3	50.0	16.7—83.3
Never	3	50.0	16.7—83.3
Alcohol			
1 per day	1	16.7	1.9—55.8
None	5	83.3	44.2—98.1
Tuberculosis history			
New	2	33.3	7.7—71.4
Prior drug-sensitive tuberculosis	4	66.7	28.6—92.3
HIV status			
HIV positive	5	83.3	44.2—98.1
HIV negative	1	16.7	1.9—55.8
CD4 count at initiation of DR-TB therapy, median (IQR) [1]		77 cells/mm^3^	26.7—154.2
ART status at diagnosis of DR-TB			
Receiving ART [2]	5	100.0	67.0—100.0

1. One of 5 HIV positive patient did not have baseline CD4 cell count.

2. Among 5 HIV positive patients participating in qualitative component of the OASIS Study.

HIV: human immunodeficiency virus; IQR: interquartile range; DR-TB: drug-resistant tuberculosis.

**Table 2:** Key aspects of person-centered, decentralized DR-TB care model compared to centralized public health approach DR-TB care model

Characteristic	Decentralized, person-centered DR-TB care	Centralized, public health approach DR-TB care
**Atmosphere**	Attentive, personalized and non-stigmatizing	Impersonal with stigmatizing experiences
**Size**	Small patient cohort, uncrowded spaces	Large patient cohort, crowded spaces
**Clinicians**	Single, consistent	Multiple, inconsistent
**Pharmacy**	Single pharmacy providing ART and DR-TB medicines with one queue	Separate pharmacy visits required for DR-TB and HIV medications
**HIV services**	HIV and DR-TB managed at one visit	HIV and DR-TB services not consolidated
**Time investment per visit**	Small time investment, limited queuing	Multiple queues with substantial time investment
**Health literacy**	Disease and treatment literacy emphasized	Health literacy not a central goal
**Patient support**	Access to on-site social worker, active patient tracing	Few patient support services

## Data Availability

The datasets used and/or analysed during the current study available from the corresponding author on reasonable request.

## Declarations

### Conferences

This work was not previously reported at a conference or meeting.
